
JOURNAL INFORMATION
==============================
NLM Title Abbreviation: Gastroenterol Rep (Oxf)
Journal ID: Gastroenterol Rep (Oxf)
NLM Journal ID: 101620508
Journal ID: gastro

Gastroenterology Report

EISSN: 2052-0034
Publisher: Oxford University Press

ARTICLE INFORMATION
==============================
PMCID: PMC5806414
DOI: 10.1093/gastro/goy004
Article ID: goy004
Article version: 1
Subjects: Chinese Abstracts

Chinese Abstracts – Volume 6 Issue 1*

Print publication date: 2018 Feb
Electronic publication date: 2018 Feb 7
Volume: 6
Issue: 1
First page: C1
Last page: C3
Copyright: © The Author(s) 2018. Published by Oxford University Press and Sixth Affiliated Hospital of Sun Yat-Sen University
Copyright year: 2018
License: This is an Open Access article distributed under the terms of the Creative Commons Attribution License (http://creativecommons.org/licenses/by/4.0/), which permits unrestricted reuse, distribution, and reproduction in any medium, provided the original work is properly cited.
License URL: https://creativecommons.org/licenses/by/4.0/

Page count: 3

==============================
Gastroenterology Report, 2018, 1–3

doi: 10.1093/gastro/goy004

ABSTRACTS

### Chinese Abstracts – Volume 6 Issue 1*

### Review（综述）

### Dysbiosis of gut microbiota in promoting the development of colorectal cancer
### 肠道菌群失调促进结直肠癌的发生

[摘要] 肠道菌群包含至少 100 万亿的细菌，定居在人类的肠道黏膜表面。最近研究显示, 肠道微生态的改变会影响正常生理, 与许多疾病相关, 包括结直肠肿瘤的发生。结直肠癌是第三常见的恶性肿瘤, 呈现多基因、多步骤的演进, 但肠道微生态在结直肠癌发生中的具体机制尚知之甚少。随着宏基因组学等新技术的出现, 使针对肠道菌群的大样本基因全貌和代谢模式分析成为可能, 从而使我们可以深入了解肠道菌群组成、分类变化及其与宿主的相互作用。不同菌属及其代谢产物在结直肠癌的发生中至关重要。同时，肠道菌群对于结直肠癌发生发展过程中的炎性反应和免疫逃逸也有重要影响。本文就目前针对肠道菌群与结直肠癌关系的研究进行了综述，从而为结直肠癌的治疗策略提供了新的视角。

关键词：肠道菌群；菌群失调；结直肠癌；肿瘤发生

### Interventions to improve physical function and prevent adverse events in cirrhosis
### 改善肝硬化患者生理功能和预防不良事件的干预措施

[摘要] 肝硬化常会并发一些致人虚弱的并发症，严重影响患者生理功能, 大大降低了患者生活质量。由于肝硬化患者出现肌肉减少症、营养不良及肝性脑病的概率较高, 因此, 功能损害或虚弱是其常见并发症。虚弱又会进一步增加患者住院治疗、意外跌倒和骨折、甚至死亡的风险。对虚弱及其相关不良反应的积极干预对于改善肝硬化患者的总体预后是非常必要的。治疗的关键是通过合理的营养和锻炼改善生理功能。补充蛋白质的营养治疗的益处已被证实， 但对于锻炼的作用一直存在争议。最新的研究结果倾向于肯定锻炼对于提高生活质量的获益。在肝病管理时的技术支持已显示出良好的应用前景。Fitbits 手环及其他可佩戴装置有助于记录和指导患者的锻炼过程和营养搭配。此外， 用于诊断和监测肝硬化并发症的智能手机软件的不断开发, 可以为肝硬化患者提供更加精细的 管理。

关键词：肝性脑病；虚弱；肌肉减少症；营养不良；肝硬化

### Treatment of recurrent *Clostridium difficile* colitis: a narrative review
### 复发性艰难梭菌结肠炎的治疗：叙述性综述

[摘要] 艰难梭菌是一种革兰阳性、能形成芽胞的专性厌氧杆菌，最初是于1935年从一名健康新生儿的粪便中分离出来的。在高收入国家， 艰难梭菌是住院患者中感染性腹泻的最常见原因。自 2000 年, 美国艰难梭菌感染的发生率快速增高, 2000 年至 2010 年间, 非孕成年人因艰难梭菌感染的住院率增加了一倍。有 20%~35% 的艰难梭菌感染患者初始抗生素治疗无效，其中有 40%~60% 的患者会出现二次复发。艰难梭菌感染在初始治疗后复发会导致许多并发症， 已成为医药卫生系统的巨大负担。本文对复发性艰难梭菌结肠炎的当前治疗进行了综述， 并对未来方向予以了展望。其治疗方式包括抗生素、益生菌、粪便移植、阴离子交换树脂、次级胆汁酸及毒素抗体。

关键词：艰难羧菌；复发性感染；粪菌移植；阴离子交换树脂；单克隆抗体；次级胆汁酸

### Original article（论著）

### Liver-inclusive intestinal transplantation results in decreased alloimmune-mediated rejection but increased infection
### 联合肝脏的小肠移植可减少免疫排斥但会增加感染

[摘要]　背景：联合肝移植被认为可以减轻其他移植器官的免疫排斥损伤， 但联合肝移植对移植小肠的影响目前尚无详细的研究数据。本研究旨在比较联合肝脏的小肠移植（LITx）与不联合肝脏的小肠移植（LETx）免疫排斥情况、移植失败的原因及临床结局。

方法：2000 年5月至 2010 年 5 月间, 212 例成人小肠移植患者纳入研究, 其中接受 LITx 者 76 例, 接受 LETx 者 136 例。 LITx 包括肝肠联合移植和含肝脏在内的多器官联合移植, LETx 则包括单独小肠移植和不含肝脏的改良多器官联合移植。

结果：在（44.9 ± 31.4）月的术后随访期间，LITx 组患者1、3、5 年移植小肠存活率分别为96.9%、93.2% 和 89.9%，LETx分别为 91.4%、69.3% 和 60.0%，两组差异有统计学意义（P<0.0001）。由于免疫排斥导致移植失败的比例 LETx 组高于 LITx 组（30.9% *vs* 6.6%, P<0.0001), 而由于感染导致移植失败的比例　LITx　组则相对较高（25.0%　*vs*　5.1%, P<0.0001）。按免疫排斥的类型进行分析，尽管两组急性细胞排斥反应的总体情况相当, 但 LITx 组发生频率（平均 0.87 *vs* 1.42, P=0.02）和严重

*This article abstract was originally published in English. This translation has not been verified and should not be relied upon—it is provided for reference purposes only. The Publishers have not checked this translation and accept no liability for completeness or accuracy of this translation or the use of this translation for whatever purpose. This translation may be incomplete and inaccurate in whole or in part. If you need to rely upon a translation of this abstract, a professional human translator should be engaged to supply an accurate translation of the original English. When referencing articles from this journal, please always refer to the original English version, rather than a translated equivalent.
本文摘要最初是以英文发表。中文摘译未获认证，谨供参考。出版社没有对中文摘译进行核验，不负责译文的完整性和准确性，也不会将其用以其他目的。中文摘译可能会出现部分不完整或不准确的情况。如果你需要准确获悉摘要，最好能请一位专业译者针对英文原文进行翻译。阅读杂志文章，请参考英文原文，不推荐中文翻译。

程度（重度比例7.9% *vs* 21.3%, P=0.01）显著优于LETx组；急性抗体介导的排斥反应发生率（3.6% *vs* 25.1%, P=0.03）和慢性排斥反应发生率（3.9% *vs* 24.3%, P=0.0002）LITx 组亦明显低于 LETx 组。

结论：联合肝脏的小肠移植似乎可以减轻术后免疫排斥, 但同时也增加了感染的风险。我们的研究结果提示, LITx 有其独特的免疫特征和临床进程, 对于行 LITx 的患者, 应降低免疫抑制治疗的强度以减少感染的发生风险。

关键词：小肠移植；免疫抑制；排斥；感染

### Ulcerative Colitis Endoscopic Index of Severity (UCEIS) versus Mayo Endoscopic Score (MES) in guiding the need for colectomy in patients with acute severe colitis

### 溃疡性结肠炎内镜下严重度指数（UCEIS 评分）与 Mayo 内镜评分（MES）用于预测急性重症结肠炎患者行肠切除术的比较研究

[摘要] 背景 溃疡性结肠炎内镜下严重度指数（UCEIS）和 Mayo 内镜评分（MES）都是溃疡性结肠炎（UC）内镜下严重程度的客观评估方法, 然而, 对于这两种评分方法能否用于筛选出需要行结肠切除术的急性重症结肠炎患者, 目前仍不明了。

方法：对 2012 年1月至 2016 年 5 月间连续收治的 ASC 患者进行回顾性分析。提取并分析入组患者的人口统计资料、既往治疗史、疾病特征资料、实验室指标、药物治疗及内镜评估等资料。主要结局指标是住院和随访期间需行结肠切除术的患者比例。

结果：92 例患者纳入研究, 其中37例（40.2%）需行结肠切除术。多因素分析显示, UCEIS 评分是需行结肠切除术的独立预测因素（OR=3.25, 95%CI：1.77~5.97；P<0.001）。UCEIS 评分的 ROC 曲线下面积为0.85；以7分作为最佳阈值, 其预测需行结肠切除术的敏感度为60.3%, 特异度为 85.5%。UCEIS 评分对于需要行结肠切除术的预测价值优于 MES 评分, 后者曲线下面积仅为 0.65。当 UCEIS 评分≥7分时, 有 80% 的 ASC 患者最终需行结肠切除术。

结论：预测ASC患者的肠切除风险, UCEIS 评分优于 MES 评分。对于 USEIS≥7 分的患者, 由于其药物治疗失败风险较高, 因此, 可考虑早期行结肠切除术。

关键词：结肠切除术；溃疡性结肠炎；内镜评估；随访

### The efficacy of rapid on-site evaluation during endoscopic ultrasound-guided fine needle aspiration of pancreatic masses

### 快速现场评估应用于内镜超声引导下细针穿刺对胰腺肿块的诊断效能

[摘要] 背景：内镜超声（EUS）引导下细针穿刺（FNA）凭借其微创性和高准确率, 已然成为胰腺肿块的最佳诊断方法。许多研究显示, 快速现场评估（ROSE）可以提高 10%~30% 的诊断率；然而, 最近的研究认为, 无需 ROSE, EUS-FNA 亦可获得满意的诊断准确率。本研究旨在评估在每次 FNA 取材后常规进行 ROSE 是否可以提高 EUS-FNA 对胰腺肿块的诊断效能。

方法：我们进行了一项回顾性研究, 研究对象是 2011 年2月至2014年10间接受EUS-FNA检查的胰腺肿块患者。所有检查均由 Emory 大学附属医院三名内镜医生完成, 均行 ROSE 操作。对纳入患者的人口统计资料、胰腺肿块的影像学特征及病理活检报告进行分析。

结果：共计 171 例患者 184 次 EUS-FNA 检查纳入分析。最终病理报告证实 128 例（70%）为恶性肿瘤。128 例恶性肿瘤中, 只有 64 例（50%）在 ROSE 下得到诊断。在这 64 例中, 有 23% 的 ROSE 需要 5 次以上（含 5 次）的 FNA 取材才发现恶性细胞。

结论：在对胰腺肿块进行 EUS-FNA 检查中应用 ROSE 可以提高诊断获益, 因为恶性细胞通常在多次FNA取材后才会被发现, 过早终止取材容易漏诊。此外, 目前针对 ROSE 的样本准备不甚理想, 只有 50% 的恶性细胞通过 ROSE 被发现。

关键词：内镜超声；细针穿刺；快速现场评估；胰腺肿块

### Effects of lifestyle modification on liver enzyme and Fibroscan in Indian patients with non-alcoholic fatty liver disease

### 生活方式调节对印度非酒精性脂肪肝病患者肝酶及 Fibroscan 扫描值的影响

[摘要] 背景：在印度等发展中国家, 随着肥胖人群的逐渐增多, 非酒精性脂肪肝病（NAFLD）的发病率快速增高。生活方式调节被推荐用于 NAFLD 患者的治疗。既往大多研究都是采用肝活检标本的纤维化分析, 来评估生活方式调节治疗后病情的改善情况。但我们无法对每一例 NAFLD 患者都进行肝活检。NAFLD 患者的肝纤维化也可以通过 Fibroscan（一种肝纤维化扫描仪）这种非侵入性的方法来评价。本研究旨在评估生活方式调节治疗对 NAFLD 患者肝酶及 Fibroscan 扫描值的影响。

方法：最初前瞻性纳入了 50 例 NAFLD 患者, 但在治疗 6 个月随访时, 只有 39 例患者回来复诊。患者分别于首次就诊和随访复诊时接受 Fibroscan 扫描。每次就诊时都对所有患者进行细致的病史采集、人体测量检查、影像及生化检查。

结果：生活方式调节治疗 6 个月后, Fibroscan 扫描值显著改善 [(7.87 ± 0.12) kPa *vs* (8.31 ± 0.11) kPa, P=0.009]；丙氨酸氨基转移酶（ALT）也显著降低[(66.69 ± 3.95) U/L *vs* (97.25 ± 2.62) U/L, P<0.001]。

结论：通过 Fibroscan 扫描值和肝酶数据可以看到, 生活方式调节可有效改善 NAFLD 患者肝损伤程度。作为一种非侵入性的检查方法, Fibroscan 可用于评估 NAFLD 患者的治疗反应。

关键词：非酒精性脂肪肝病；生活方式调节；丙氨酸氨基转移酶；Fibroscan 肝纤维化扫描仪

### Comparative analysis of different hepatico-jejunostomy techniques for treating adult type I choledochal cyst

### 不同胆管空肠吻合技术治疗成人 I 型胆总管囊肿的比较分析

[摘要] 目的：比较囊肿完全切除与不完全切除（残留近端1 cm胆总管囊壁）的 Roux-en-Y 胆管空肠吻合治疗成人 I 型胆总管囊肿的安全性和可行性。

方法：回顾性分析 1998 年1月至 2015 年 12 月间收治的 267 例成人 I 型胆总管囊肿患者的病例资料, 其中 171 例行囊肿完全切除的 Roux-en-Y 胆管空肠吻合术（PBD 0-cm 组）, 96 例行囊肿不完全切除的 Roux-en-Y 胆管空肠吻合术（PBD 1-cm 组）。比较两组患者术后短期及长期并发症发生情况。

结果：两组患者在手术时间和吻合口直径方面的差异均无统计学意义。PBD 1-cm 组围手术期并发症发生率显著高于 PBD 0-cm 组（28.1% *vs* 14.0%, P=0.005）, 尤其是术后胆管炎（7.3% *vs* 1.2%, P=0.021）。两组患者术后吻合口狭窄、反流性胆管炎、肝内胆管结石和胆漏等长期并发症的差异均无统计学意义（均 P>0.05）。PBD 1-cm 组和 PBD 0-cm 组各有1例患者分别于术后5月和术后 25 月出现胰胆管恶变。而 PBD 1-cm 组有1例患者于术后 10 月出现吻合口恶变。

结论：虽然保留部分囊壁可以降低 I 型胆总管囊肿切除术中的吻合难度, 但由于相对较高的术后并发症发生率和恶变概率, 该吻合方式值得商榷。对于 I 型胆总管囊肿患者, 囊肿完全切除后以健康的近端胆管进行吻合是必要的。

关键词：胆总管囊肿；Roux-en-Y 胆管空肠吻合术；胆汁引流；成年人

### Variations in the medical treatment of inflammatory bowel disease among gastroenterologists

### 胃肠科医生对炎症性肠病药物治疗的差异

[摘要] 背景与目的：随着炎症性肠病（IBD）治疗药物选择的不断增多以及对药物治疗风险和获益认识的不断深入, IBD 患者的治疗方式和临床结局可能存在显著的差异。对于 IBD 专科医生与其他胃肠科医生之间的治疗差异, 目前所知甚少。评估这种可能的差异是开展规范化治疗和

优化治疗方式的重要前提。我们分析了一家三甲医院 IBD 专科医生与其他胃肠科医生进行 IBD 生物治疗和免疫治疗中存在的差异。

方法：共计 325 名 IBD 患者纳入研究，其中 216 例接受 IBD 专科医生的治疗，另外 109 例由非 IBD 专科的胃肠科医生担任他们的主治医生。

结果：由 IBD 专科医生施治的患者，其接受免疫抑制剂（35.6% *vs* 16.5%, $P$=0.001）、生物制剂（45.8% *vs* 22.9%, $P$=0.001）和双药联合治疗（14.4% *vs* 3.7%, $P$=0.001）的比例明显高于在其他胃肠科医生就治的患者。在对患者及疾病特征进行调整后，这些差异仍然存在。

结论：不同的胃肠科医生对于 IBD 患者的治疗策略存在显著差异，其中 IBD 专科医生更倾向于采用生物治疗和免疫治疗。

关键词：治疗差异；炎性肠病；克罗恩病；溃疡性结肠炎；生物制剂；免疫抑制剂

### Case report（病例报告）

### Vogt-Koyanagi-Harada syndrome and Crohn's disease: an exceptional association

Vogt-Koyanagi-Harada 综合征与克罗恩病：一种独特的关系

### Severe cholestatic hepatitis due to large vessel vasculitis: report of two cases

大血管的血管炎导致重度淤胆型肝炎：两例报告

### Splenic tuberculosis in an immunocompetent patient can be managed conservatively: a case report

免疫功能正常的脾结核患者可保守治愈：一例报告
